# Real-time ultrasound-guided versus anatomic landmark-based thoracic epidural placement: a prospective, randomized, superiority trial

**DOI:** 10.1186/s12871-022-01730-5

**Published:** 2022-06-25

**Authors:** Jatuporn Pakpirom, Kanthida Thatsanapornsathit, Nalinee Kovitwanawong, Suttasinee Petsakul, Pannawit Benjhawaleemas, Kwanruthai Narunart, Somrutai Boonchuduang, Manoj Kumar Karmakar

**Affiliations:** 1 Department of Anesthesiology, Faculty of Medicine, Prince of Songkla University, Hat Yai, Songkhla, 90110 Thailand; 2 Department of Anaesthesia and Intensive Care, Faculty of Medicine, The Chinese University of Hong Kong, Prince of Wales Hospital, Shatin, New Territories, Hong Kong, SAR China

**Keywords:** Analgesia, Anesthesia, Landmark-based technique, Thoracic epidural placement, Ultrasound-assisted, Ultrasonography

## Abstract

**Background:**

Thoracic epidural placement (TEP) using the conventional anatomic landmark-based technique is technically challenging, may require multiple attempts, and is associated with a high failure rate (12–40%). We hypothesized that real-time ultrasound guidance would be superior in the “first-pass” success rate of TEP, when compared with the conventional technique.

**Methods:**

This prospective, randomized, superiority trial was conducted in a University hospital, and recruited 96 patients undergoing elective major abdominal or thoracic surgery and scheduled to receive a TEP for postoperative analgesia. Patients were randomly allocated to receive TEP using either the conventional technique (Gp-Conv, *n* = 48) or real-time ultrasound guidance (Gp-Usg, *n* = 48). The success of TEP was defined as eliciting loss of resistance technique and being able to insert the epidural catheter. The primary outcome variable was the “first-pass success rate” meaning the successful TEP at the first needle insertion without redirection or readvancement of the Tuohy needle. The secondary outcomes included the number of skin punctures, number of attempts, the overall success rate, TEP time, and total procedure time.

**Results:**

The first-pass success rate of TEP was significantly higher (*p* = 0.002) in Gp-Usg (33/48 (68.8%); 95%CI 55.6 to 81.9) than in Gp-Conv (17/48 (35.4%); 95%CI 21.9 to 49.0). There was no statistically significant difference (*p* = 0.12) in the overall success rate of TEP between the 2 study groups (Gp-Usg; 48/48 (100%) vs. Gp-Conv; 44/48 (91.7%); 95%CI 83.9 to 99.5). Ultrasound guidance reduced the median number of skin punctures (Gp-Usg; 1 [1, 1] vs Gp-Conv; 2 [1, 2.2], *p* < 0.001) and attempts at TEP (Gp-Usg; 1 [1, 2] vs Gp-Conv; 3 [1, 7.2], *p* < 0.001) but the procedure took longer to perform (Gp-Usg; 15.5 [14, 20] min vs Gp-Conv; 10 [7, 14] min, *p* < 0.001).

**Conclusions:**

This study indicates that real-time ultrasound guidance is superior to a conventional anatomic landmark-based technique for first-pass success during TEP although it is achieved at the expense of a marginally longer total procedure time. Future research is warranted to evaluate the role of real-time ultrasound guidance for TEP in other groups of patients.

**Trial registration:**

Thai Clinical Trials Registry; http://www.thaiclinicaltrials.org/; Trial ID: TCTR20200522002, Registration date: 22/05/2020.

## Introduction

Thoracic epidural analgesia (TEA) is the established gold standard for postoperative pain management after major open thoracic and abdominal surgery [1–3]. However, although TEA improves postoperative outcomes [1–3] access to the thoracic epidural space using the conventional anatomic landmark-based technique (henceforth referred to as conventional technique) can be technically challenging [4]. This is more so in the mid-thoracic region (T5-T9) where the interlaminar spaces are relatively narrow [5, 6], due to the laminae being closely stacked up against each other, and the spinous processes are steeply angled caudally [6]. Moreover, age-related changes in the anatomy of the thoracic spine, e.g. closure of the interlaminar spaces and calcification of the supraspinous ligament, can also limit access to the epidural space [6]. Consequently, the conventional technique of thoracic epidural placement (TEP), whereby the needle is frequently inserted using the paramedian approach, may require multiple attempts [7], and is associated with a relatively high failure rate (12–40%) [4, 8]. Serious technical complications after TEP are relatively rare (1:1000, [9]) but can lead to needle misadventure with spinal cord injury [10], placement of a catheter into the ipsilateral [11] or contralateral [12] pleural cavity, and even pneumothorax [13]. Therefore any technique that can improve technical precision, reduce the number of attempts, and improve the safety of TEP is desirable.

Ultrasonography of the lumbar [14, 15] and thoracic spine [14, 16–18] is currently feasible and has been used to facilitate central neuraxial blocks [19–21] including TEP [8, 16, 17, 22]. When used in the thoracic region it is used either as a preprocedural ultrasound scan [16, 22] or to guide the epidural needle in real-time [8, 17]. With the preprocedural ultrasound scan one can accurately determine a given thoracic intervertebral level [16, 23], identify the mid-line [16], determine the best needle insertion point and trajectory for needle insertion [24], and measure the depth to the epidural space [16, 22, 23, 25]. However, a preprocedural ultrasound scan on its own doesn’t appear to improve the technical precision of TEP or reduce the time required to identify the thoracic epidural space when compared with the conventional technique [16]. Also, although real-time ultrasound-guided TEP is technically feasible [8, 17] and accurate (76% first-pass success) [8] there is a paucity of data on the technique [8, 17] and no randomized controlled trials comparing real-time ultrasound guidance with the conventional technique. In this study, we hypothesized that real-time ultrasound guidance would improve the “first-pass” success rate of TEP, when compared with the conventional technique.

## Methods

**Fig. 1:**
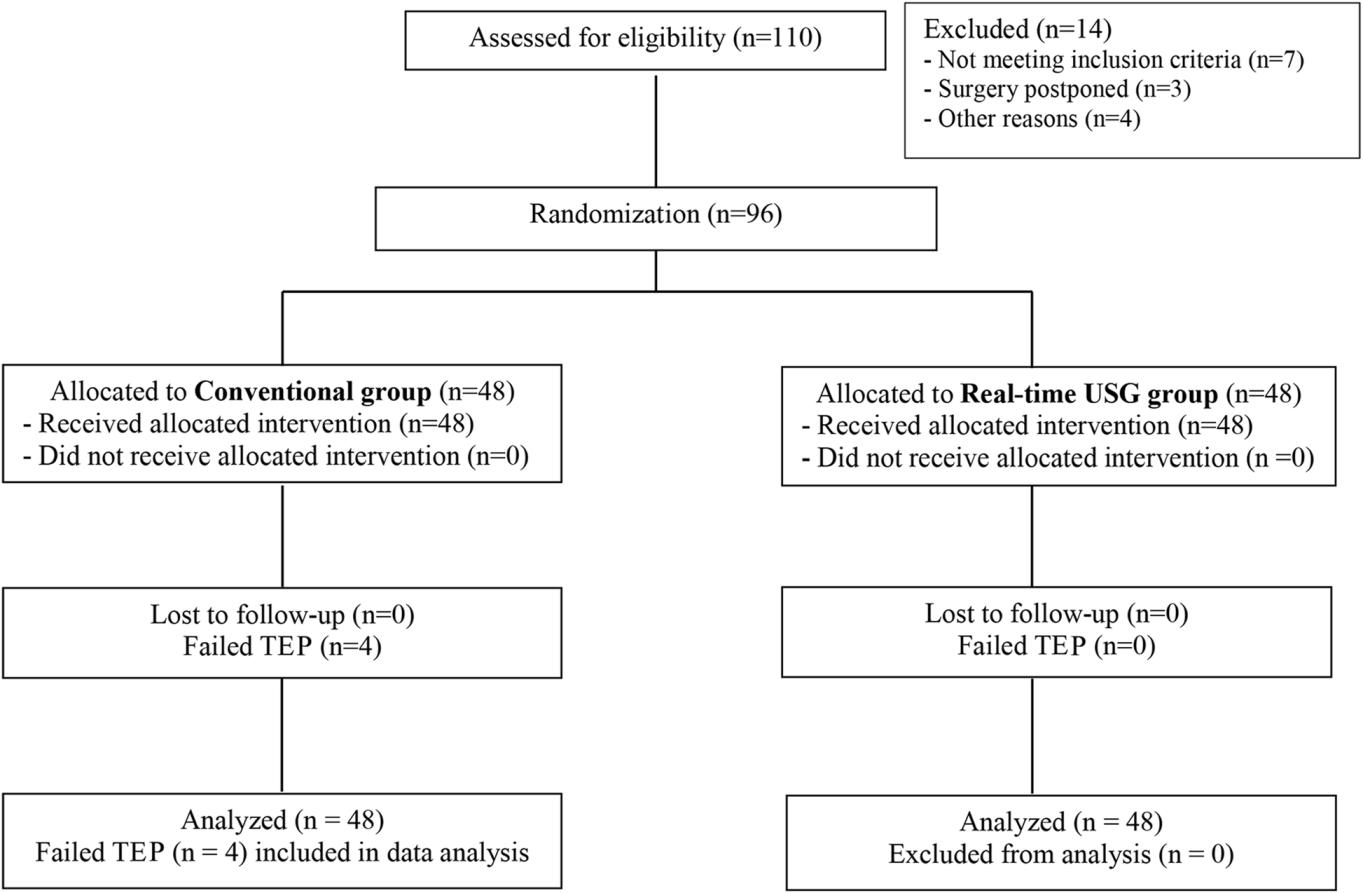
Consort flow chart

This prospective, randomized, single-center, study was approved by the Human Research Ethics Committee of the Faculty of Medicine, Prince of Songkla University, Thailand on 2 March 2020, and it was conducted in accordance with the relevant guidelines and regulations by the ethical committee. This study was prospectively registered with the Thai Clinical Trials Registry (http://www.thaiclinicaltrials.org/) under the Trial ID: TCTR20200522002 (Registration date 20/05/2020; Issue date 22/5/2020). Patient enrollment date on 22 May 2020–15 February 2021. Of the 110 adult patients who were screened for recruitment, 96 patients who gave written informed consent and were scheduled to undergo elective thoracic or abdominal surgery with a planned postoperative TEA at Songklanagarind hospital (University setting) were recruited (Fig. 1). Exclusion criteria included the following: patient’s refusal, pregnancy, contraindications for central neuraxial blocks, morbid obesity (BMI > 35 kg/m^2^), allergy to local anesthetic drugs, presence of spinal deformity, and history of thoracic spine surgery.

### Randomization

Patients were randomized into either Gp-Conv (conventional technique, *n* = 48) or Gp-Usg (real-time ultrasound-guided, *n* = 48) for the TEP by drawing sequentially numbered, coded, sealed opaque envelopes that contained a card with a computer-generated allocation number (1 = Gp-Conv, 2 = Gp-Usg). The randomization sequence and envelopes were prepared by a third party who took no further part in the study. The surgeons, investigators and outcome assessors were all unaware of the randomization sequence before the TEP.

### Pre-operative preparation

At the preoperative visit, patients were explained about postoperative TEA and invited to participate in the study. Written informed consent was obtained from those who agreed to participate in the study. All patients were fasted and prescribed no sedative drug premedication. The TEP was performed in a procedure room about 1–1.5 h before the scheduled surgery. On arrival, a regional anesthesia preprocedural checklist was applied and standard monitoring was instituted. Patients were also instructed on how to rate the discomfort experienced during the TEP using a verbal numeric rating scale (0–10, 0 = comfort, 10 = worst imaginable discomfort). The oxygen supplementation (1–2 l/min) was commenced and midazolam (1–2 mg) and/or fentanyl (50 μg) were administered intravenously for procedural sedation.

### Blinding method

The anesthesiologist performing the TEP, and the outcome assessor (nurse assistant) could not be blinded to group allocation because the nurse had to record the procedural data. Patients were unaware of the group allocation and were all placed in the lateral decubitus position for the TEP. The ultrasound machine was placed in front of the patient and the anesthesiologist was positioned behind the patient. Irrespective of the study group ultrasound was used to locate the target thoracic intervertebral level by identifying the ribs space as described below. In Gp-Conv, the ultrasound scan was stopped and frozen at the intercostal view (to semi-blind the patients) while in Gp-Usg real-time ultrasound images were continuously displayed. So in Gp-Conv ultrasound imaging was used only to identify the thoracic intervertebral level and the epidural catheterization was performed using the traditional palpation (tactile) and loss-of-resistance technique. In contrast, in Gp-USG ultrasound was used to locate the thoracic intervertebral level, preview the spinal anatomy, identify the target intervertebral, and guide the epidural needle in real-time to the ligamentum flavum, after which loss-of-resistance was used to identify the epidural space before catheter placement.

### Thoracic epidural placement

**Fig. 2:**
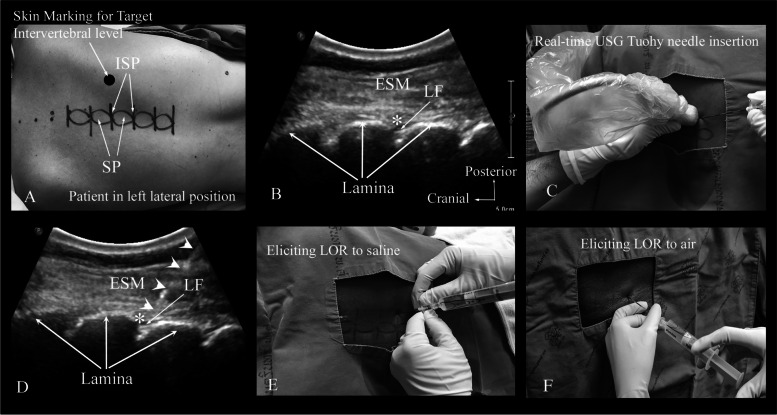
The technique of real-time ultrasound-guided thoracic epidural catheterization. **A** Patient positioned in the left-lateral decubitus position. Note the skin markings of the thoracic spinous processes (SP) and interspinous spaces (ISS) at the target thoracic level. **B** Paramedian sagittal oblique sonogram of the target intervertebral level showing the laminae and interlaminar spaces. **C** Paramedian, in-plane, real-time ultrasound-guided (USG) Tuohy needle insertion from the caudal end of the transducer and from the non-dependent side. **D** Paramedian sagittal oblique sonogram demonstrating the Tuohy needle (white arrowheads) insertion in-plane and with its tip located adjacent to the interlaminar space, (**E**) Eliciting loss-of-resistance (LOR) to injection of saline to locate the epidural space. Note how the hands of the operator support the Tuohy needle—LOR syringe assembly on the patients back, (**F**) Illustrating LOR to air to locate the epidural space. SP; indicates a spinous process, ISP; interspinous space, ESM; erector spinae muscle, LF; ligamentum flavum, _*_ represents the interlaminar space

In Gp-Conv the TEP was performed by an attending anesthesiologist (specialist experience ranged from 4 to 15 years’). In Gp-Usg the TEP was performed by one of three anesthesiologists (JP, PB and KN) (with 2 to 5 years post-fellowship experience in ultrasound-guided regional anesthesia). The TEP was performed under aseptic precautions and with the patient in either left or right lateral decubitus position in Gp-Conv. However, in Gp-Usg, all patients were positioned in the left lateral (Fig. 2A), to improve the dexterity of the operator using the dominant (right) hand for needling while performing the TEP. The target thoracic intervertebral level used for the TEP depended on the type of surgery and the preference of the anesthesiologists.

**Fig. 3:**
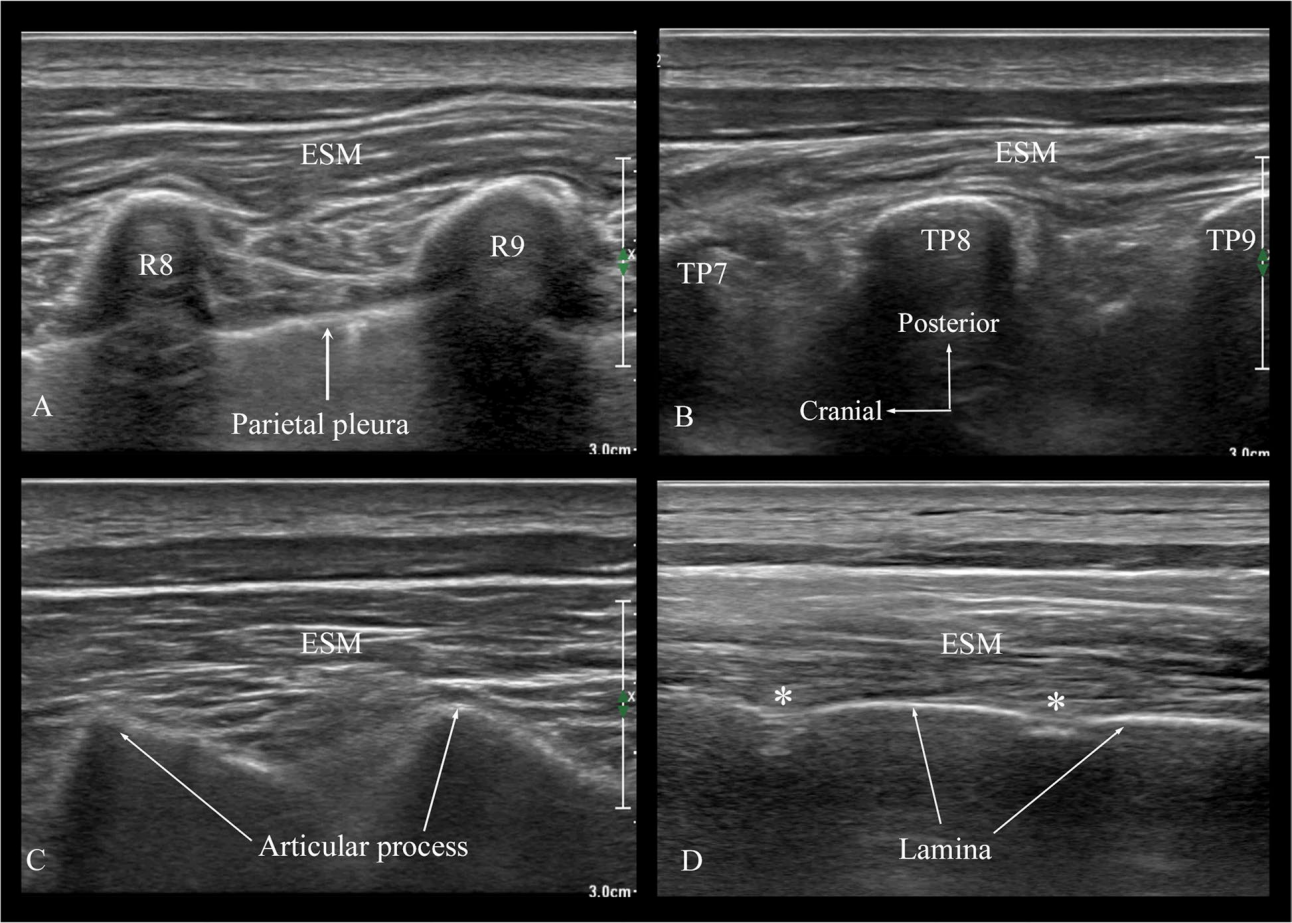
Ultrasound sequence to identify the relevant sonoanatomy at the target intervertebral level for the real-time thoracic epidural catheterization. **A** Paramedian sagittal sonogram at the level of the 8^th^ intercostal space after counting up from the 12.^th^ rib. Note the rounded ribs and the hypoechoeic pleura. **B** Paramedian sagittal sonogram at the level of the transverse process (TP). Note the pleural is less clearly visualized at the level of the transverse processes. **C** Paramedian sagittal sonogram at the level of the articular processes. Note the sonographic appearance of the articular processes varies from that of the ribs and transverse processes. **D** Paramedian sagittal oblique sonogram at the level of the laminae of the thoracic vertebra, Note the flattened appearance of the lamina. R; represents the rib, TP; transverse process, ESM; erector spinae muscle, _*_ represents the interlaminar space

A Phillips Affiniti 50 ultrasound system (Philips Healthcare, Andover, MA) with a low-frequency curvilinear (3–6 MHz) or a high-frequency linear (5–12 MHz) transducer was used during the study. The target intervertebral level for the TEP was confirmed by using ultrasound by performing a paramedian sagittal scan (5–7 cm away from the midline) at the lower thoracic cage to locate the 12^th^ rib and then counting upwards [25] to identify the intercostal space at the target intervertebral level (Fig. 3A).

A Perifix® epidural set (B-Braun, Germany), with an 18 gauge Tuohy needle and a 20 gauge multi-orifice catheter, was used for the TEP in both study groups. After successfully locating the epidural space 4–5 cm of the epidural catheter was advanced into the epidural space, and it was then safely tunneled subcutaneously, secured, and covered using a clear transparent dressing (3 M™ Tegaderm™, USA).

### Conventional TEP (Gp-Conv)

The TEP was performed using either the midline or the paramedian approach. For the midline approach, the Touhy needle was inserted at the interspinous space, and for the paramedian approach the needle was inserted 1 cm below the midline. After skin infiltration, the Touhy needle was inserted and advanced in a caudo-cranial direction until the needle entered the ligamentum flavum. The epidural space was identified using loss-of-resistance (LOR) to saline or air (Fig. 2 E and F), depending on the choice of the anesthesiologists.

### Real-time ultrasound-guided TEP (Gp-Usg)

The ultrasound transducer with its cable was covered using a sterile plastic sleeve (Fig. 2C). Normal saline was used instead of the ultrasound gel as the coupling agent to prevent the unintentional introduction of ultrasound gel into the epidural space during the TEP. The real-time USG TEP was performed from the non-dependent side, with the patient in the left lateral position (Fig. 2C), and in three sequential steps. Step 1. Identification of the interlaminar space at the target intervertebral level. (Fig. 2B) From the sagittal scan at target rib space (Fig. 3A), the ultrasound transducer was then slid medially, maintaining the same sagittal orientation, until the transverse processes of the thoracic vertebra were visualized (Fig. 3B). Further sliding of the transducer medially brought the articular processes into view in the sagittal sonogram (Fig. 3C), the transducer was then gently tilted medially (paramedian sagittal oblique scan) until the lamina and the interlaminar spaces were identified (Figs. 2B and 3D). Step 2. With the target interlaminar space in view on the ultrasound monitor (Fig. 2B), the skin was infiltrated at the caudal end of the ultrasound transducer. The Tuohy needle was then inserted in-plane and advanced under real-time ultrasound guidance and in a caudo-cranial direction (Fig. 2C) until the needle tip was seen to lie adjacent to the target interlaminar space (Fig. 2D) or judged to have engaged in the ligamentum flavum. Step 3. With the Tuohy needle tip engaged in the ligamentum flavum the ultrasound transducer was removed and the LOR syringe was attached to the Touhy needle hub (Fig. 2E and F). The needle-syringe assembly was then slowly advanced, and the LOR to normal saline was intermittently elicited to confirm entry of the needle tip into the epidural space. If there was a bony contact, minimal needle manipulation was performed to enter the epidural space. However, if it was not possible to locate the epidural space due to multiple bony obstructions (> 3 attempts) then the needle-syringe assembly was completely withdrawn and the ultrasound transducer was reapplied to evaluate the sonoanatomy. Thereafter the procedure was repeated at the same interlaminar space or an adjacent interlaminar space was used.

### Intraoperative/postoperative TEA management

After negative aspiration of the epidural catheter, a test dose of 3 ml of 2% lidocaine with epinephrine was injected, and vital parameters were monitored at 3 min’ intervals. Loss of sensation to pin-prick was used to assess the extent of sensory blockade after the test dose, and to confirm correct thoracic epidural placement. Patients were then transferred to the operating room and a standardized general anesthetic was administered for surgery. Once patients were hemodynamically stable an additional 5-ml bolus of a mixture of 0.2% bupivacaine and fentanyl (2 μg/ml) was slowly injected via the epidural catheter before skin incision and a continuous infusion of the same epidural mixture was commenced at 0.1 ml/kg/h. Once patients arrived in the recovery room the epidural infusion was changed to a mixture of 0.1% bupivacaine and fentanyl (2 μg/ml) at 0.1 ml/kg/h for postoperative analgesia. Postoperative TEA was managed by our acute pain team until the epidural catheter was removed.

### Outcome measures

The primary outcome of this study was the “first-pass success rate”, which was defined as successful TEP with a single skin puncture, minimal needle tip manipulation, and no needle redirections. Successful TEP was defined as being able to elicit LOR and insert a predetermined length (4 to 5 cm) of the catheter into the epidural space. If the TEP was technically difficult and the procedure took longer than 30 min, it was considered a failure. Secondary outcomes included the number of skin punctures and attempts, failure rate, preparation time, TEP time, total procedure time and any complications. For defining the number of attempts, if after the first skin puncture and Tuohy needle insertion the needle was withdrawn and re-advanced or redirected, with or without additional skin puncture, it was recorded as an additional attempt. Preparation time was recorded as the time from the start of patient positioning to the completion of skin infiltration. TEP time was defined as the time it took from Tuohy needle insertion to removal of the needle after catheter placement. The total procedure time represented a combination of the preparation time and TEP time. The discomfort experienced was rated on a verbal numeric rating scale as described above.

### Statistical methodology

#### Sample size calculation

Sample size was calculated using the n4Studies smartphone app [26]. A pilot study (*n* = 20) in our hospital demonstrated that the first-pass success rate for TEP using real-time ultrasound guidance was 60%. We assumed that the “first-pass success” rate for the conventional technique would be 50% of ultrasound-guided (30%). The sample size was calculated to test the hypothesis that real-time ultrasound guidance would be superior to a conventional technique for “first-pass success” during TEP with the superiority margin (δs) set at 0.05. It was thereby estimated that a sample size of 90 (45 patients per study group) would provide 80% power to demonstrate the difference in first-pass success rate between the two techniques with an α error of 0.05. We recruited 48 patients per study group to compensate for any potential dropouts or variability in the data recorded.

#### Data analysis

R program (The R Foundation, V.3.5.3) was used for statistical analysis, and the normality distribution of continuous variables was tested using the Shapiro–Wilk test. Data are presented as mean ± SD) or median [interquartile range] depending on the data distribution, and Student *t*-test or Wilcoxon rank-sum test were used as appropriate to compare the outcomes. Frequency n(%) was used to present categorical variables, and the *x*^*2*^ or Fisher-exact test was used to compare between the groups. *P* < 0.05 was considered statistically significant. To demonstrate the superiority of real-time ultrasound guidance over the conventional technique of TEP the proportion and 95% confidence interval (CI) of the first-pass success rates for both study groups were computed and Gp-Usg was defined as being superior to Gp-Conv if the lower bound of the 95% CI of Gp-Usg was beyond the pre-specified δs. In this study, the δs was set at 5% because we believed this would be the smallest difference that would be clinically important (clinical significance).

## Results

**Fig. 4:**
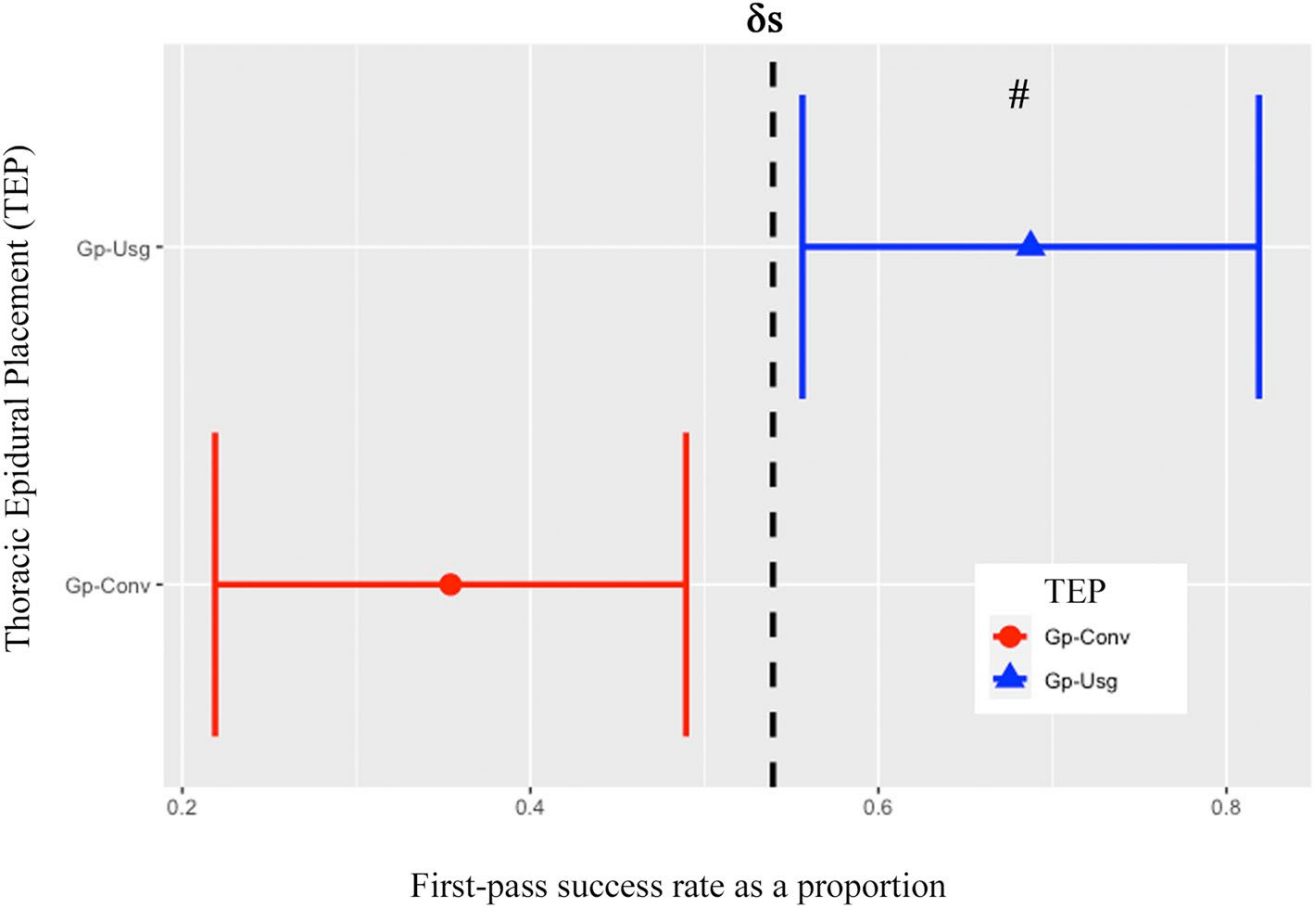
The first-pass success rate of thoracic epidural placement in the study groups. Data are presented as a proportion with its 95% CI (confidence interval). The Black dashed line represents the pre-specified superiority margin (δs = 0.05). Note the lower bound of the 95%CI for the first-pass success rate of Gp-Usg (real-time ultrasound-guided group) does not overlap the upper bound of the 95%CI of Gp-Conv (conventional anatomic landmark group) and it is beyond the pre-specified δs of 5% confirming clinical superiority of Gp-Usg. **#** represents inter-group difference *p* = 0.002, and TEP, thoracic epidural placement

Ninety-six patients were randomized to either Gp-Usg (*n* = 48) or Gp-Conv (*n* = 48) (Fig. 1). There was an overall failure of the TEA in 5(10.4) patients in Gp-Conv and none in Gp-Usg (*p* = 0.056). However, data from all patients were included in the final analysis. The 2 study groups were comparable in demographic data and clinical characteristics except that patients in Gp-Conv underwent more thoracic surgery than that in Gp-Usg (Table 1). Nevertheless, more (*p* = 0.03) TEP were performed in the mid-thoracic spine in Gp-Usg (91.7%) than in Gp-Conv (72.9%). The first-pass success rate of TEP was significantly higher (*p* = 0.002) in Gp-Usg (68.8%; 95%CI 55.6 to 81.9) than in Gp-Conv (35.4%; 95%CI 21.9 to 49) (Table 2). Also, the lower bound of the 95%CI for the first-pass success rate of Gp-Usg was beyond the pre-specified δs (5%) of the 95%CI in Gp-Conv (Fig. 4) confirming clinical superiority of Gp-Usg.

**Table 1:** Demographic Data and Clinical Characteristics of the Study Groups

	Conventional Group (*n* = 48)	Ultrasound Group (*n* = 48)	*P* value
1. Age (yrs.)	58.5 [53.75 to70.25]	60.0 [51.0 to 67.0]	0.53
2. Gender (male/female), n	28/20	26/22	0.84
3. ASA status (I/II/III), n	0/34/14	2/36/10	0.29
4. Weight (kg)	59.4 ± 11.8	60.3 ± 10.2	0.68
5. Height (cm)	162.50 [155 to 166.2]	160 [154.8 to 168.2]	0.69
6. BMI, (kg (m^2^)^−1^)	22.8 ± 3.5	23.4 ± 4.0	0.46
7. Type of surgery (a/b/c), n	23/15/10	12/27/9	0.03
8. TEP level, n			0.03
T5-T8/T9-T12	35/13	44/4	
9. Number of patient’s who	1	0	0.47
did not achieve sensory			
blockade after the test			
dose, n			
10. Number of Dermatomes	5 [4 to 7]	5 [4 to 7]	0.86
blockade after the test			
dose, n			

Data are presented as mean ± SD, median [IQR Q1 to Q3], frequency (n), type of surgery: a; thoracic surgery, b; upper abdominal surgery, c; lower abdominal surgery

*ASA* American Society of Anesthesiologist, *BMI* body mass index, *TEP* thoracic epidural placement, *mcg* microgram, *T5-T8* mid-thoracic spine, *T9-T12* lower thoracic spine

**Table 2:** Procedural data relating to the TEP in the two study groups

	Conventional Group (*n* = 48)	Ultrasound Group (*n* = 48)	*P* value
1. First-pass success;	17 (35.4)	33 (68.8)	0.002
n (%) (95% CI)	(21.9 to 49.0)	(55.6 to 81.9)	
2. TEP success^a^;	44 (91.7)	48 (100)	0.117
n (%) (95% CI)	(83.9 to 99.5)	(100 to 100)	
3. Overall TEP failure rate^b^;	5 (10.4)	0 (0)	0.056
n (%) (95% CI)	(1.77 to 19.06)	(0 to 0)	
4. Number of skin punctures	2 [1 to 2.25]	1 [1 to 1]	< 0.001
5. Number of attempts	3 [1 to 7.2]	1 [1 to 2]	< 0.001
6. Success rate of TEP with			
each attempt; n (%)			< 0.001
One attempt	17 (35.4)	33 (68.8)	
Two attempt	4 (8.4)	10 (20.8)	
Three attempt	5 (10.4)	3 (6.2)	
Four attempts	5 (10.4)	2 (4.2)	
≥ Five attempts	17 (35.4)	0 (0.0)	
7. Preparation time (min)	5 [3.8 to 6]	13.5 [11 to 15]	< 0.001
8. TEP time (min)	2 [1 to 4]	1.5 [1 to 3]	0.190
9. Total procedure time (min)	10 [7 to 14]	15.5 [14 to 20.2]	< 0.001
10. Discomfort score (0–10)	1 [0 to 5]	0 [0 to 4.2]	0.44

Data are presented as a frequency (percentage), n (%), frequency (percentage) (95% confidence interval), *n* (%) (95% CI), or median [IQR Q1 to Q3]. ^a^ the calculate according to the definition of success TEP (achieve a loss of resistance technique and able to insert the catheter), ^b^ the calculation from primary failure TEP, and included intraoperative catheter failure, *CI* confidence interval, *IQR* interquartile rang, *TEP* thoracic epidural placement

Procedural data relating to the TEP for the two study groups are presented in Table 2. The median number of skin punctures was significantly higher (*p* < 0.001) in Gp-Conv than in Gp-Usg. Patients in Gp-Usg also required fewer (*p* < 0.001) attempts for the TEP than that in Gp-Conv. 17/48(35.4%) patients in Gp-Conv required more than 5 attempts for the TEP but in Gp-Usg it was achieved in most patients (43/48(89.6%)) within 2 attempts (*p* < 0.001). There was no difference in TEP time between the 2 study groups (*p* = 0.20), but the preparation time and total procedure time were significantly longer in Gp-Usg (*p* < 0.001). There was no difference (*p* = 0.44) in the discomfort experienced between the 2 study groups. There were no complications directly related to the technique or local anesthetic injection except for one patient in Gp-Usg where accidental intravascular placement was suspected. Therefore, the catheter was removed and repositioned at a higher level.

## Discussion

In this prospective, randomized trial, we compared the first-pass success rate of TEP between real-time ultrasound guidance and the conventional anatomic landmark-based technique.

Under the conditions of this study, our findings confirm our hypothesis that real-time ultrasound guidance not only improves the technical precisions for TEP but is also clinically superior to the conventional technique for the first-pass success rate of TEP. Currently, there is a paucity of data on real-time ultrasound guidance for TEP [8, 17] and we believe this is the first study to demonstrate that real-time ultrasound guidance improves technical precision during TEP when compared to the conventional technique.

The first pass-success rate of TEP with real-time ultrasound guidance (68.8%) was significantly higher than that with the conventional technique (35.4%). Currently, there are no comparable data but our first-pass success rate of TEP in the 2 groups are comparable to that reported for conventional (42 to 45%) [27] and real-time USG TEP (73 to 76%) [8, 17]. The near 100% improvement in first-pass success for TEP in Gp-Usg compared to Gp-Conv that we have demonstrated, also confirms that our finding is clinically relevant. We believe the higher first-pass success rate in Gp-Usg was due to the greater precision with which the Tuohy needle could be advanced to the target interlaminar space before entering the epidural space was located using LOR. Currently, there are no published data comparing the clinical benefits of real-time ultrasound-guided over the conventional technique for TEP. It is also noteworthy that more TEP’s were performed in the mid-thoracic region in Gp-Usg than in Gp-Conv (Table 1). Therefore, despite the greater technical difficulty of TEP in the mid-thoracic region (T5-T8) than in the low-thoracic region (T9-T12) [5, 27] our higher first-pass success in Gp-Usg is further evidence that real-time ultrasound guided technique is superior for TEP than the conventional technique. Future research to compare the technical success rate and clinical efficacy of TEP using real-time ultrasound guidance in the mid- vs lower-thoracic levels is warranted.

The number of skin punctures and attempts at TEP was significantly lower in Gp-Usg than in Gp-Conv. There are no comparable data with TEP but Grau and colleagues [28] have demonstrated that real-time ultrasound visualization reduces needle puncture and spinal needle manipulation during combined spinal-epidural anesthesia [28]. We believe our results reflect improved precision during needle advancement with real-time ultrasound guidance. Also given that fewer skin punctures and needling attempts were required with real-time ultrasound guidance one might expect patients in Gp-Usg to experience less procedural discomfort than that in Gp-Conv. However, we found that patients in both study groups reported low procedural discomfort and tolerated the procedure well. This might be because of the procedural sedation, and the local anesthetic skin infiltration. We acknowledge that discomfort score was a secondary outcome in this study and thus not powered to demonstrate a difference in this outcome. Therefore, future randomized studies should determine if real-time USG can reduce procedural discomfort during TEP.

The TEP success rate and overall TEP failure rate in the 2 groups were similar. There are no comparable data but our technical success rates for Gp-Usg (100%) and Gp-Conv (90%) are comparable with that reported in the literature [8, 16, 17]. Nevertheless, the power of this study was not adequate to demonstrate this difference. It is also noteworthy that the total procedure time was marginally longer in Gp-Usg than that in Gp-Conv. This is understandable and reflects the additional time required in Gp-Usg to perform the ultrasound scan and aseptically prepare the ultrasound transducer. The technical success of TEP depends on multiple factors that may be related to the patient, technique or the experience of the operator. This was a randomized study and the two techniques being compared were performed by two groups of experienced anesthesiologists. Therefore, operator-related factors should not have affected the TEP success rate.

The clinical significance of the improved first-pass success rate and reduced number of skin punctures required for the TEP in Gp-Usg of this study is not entirely clear as there are no comparable data in the literature. However, a recent systematic review and meta-analysis [29] comparing the landmark-based technique with a preprocedural ultrasound scan for neuraxial anesthesia in the obstetric population found that a preprocedural ultrasound significantly increased the first-pass success rate and reduced the incidence of complications including bloody tap or vascular cannulation, and postpartum headache and backache [29]. The Authors [29] hypothesized that the reduced skin punctures and needle redirections during the neuraxial block may decrease the potential for the development of micro-hematoma in the back and thus lower the incidence of postpartum backache. Also, the reduced occurrence of bloody tap and vascular cannulation in the preprocedural ultrasound group was considered encouraging as they are often associated with the development of a spinal hematoma [30].

Our study presents several limitations. Patients were of low BMI but are consistent with the population studied at the authors’ institution. Therefore, our results may not apply to the obese population. The inherent nature of the study also made it difficult to blind the operator and the outcome assessor. The former was because we had to ensure that the TEP in both study groups was performed by an experienced operator. Therefore, it is not possible to completely exclude operator bias. The latter was because the nurse assistant had to record procedural data during the TEP. However, given that the primary outcome variable, i.e. first-pass success, had a binary response (yes or no) we believe blinding may not have affected the final results. We had considered video recording the entire TEP procedure and had a third party assess the first-pass success but felt that the outcome assessor would still be unblinded to the technique used, from watching the video, and thus may have yielded the same result. We did not use any alternative technique (epidurogram, MRI or waveform analysis) to confirm correct epidural catheter placement but tested for the presence of thoracic sensory blockade after the test dose in all patients. Finally, our results may not apply to every anaesthesiologist because the TEP in Gp-Usg was performed by regional anesthesiologists experienced in USG regional anesthesia. Despite some of these limitations, we believe our data are still valid but should be interpreted with caution until more confirmatory data are available.

## Conclusion

In conclusion, our results indicate that real-time ultrasound guidance is superior to a conventional anatomic landmark-based technique for first-pass success during TEP, although this is achieved with the expense of a marginally longer total procedure time. Future research should evaluate the role of real-time ultrasound guidance for TEP in a more heterogeneous cohort of patients and when performed by operators with varying levels of experience.

## Data Availability

Data are available on request from the first author (JP).

## Abbreviations

ASA: American Society of Anesthesiologist
BMI: Body mass index
CI: Confidence interval
CSF: Cerebrospinal fluid
Gp-Conv: Conventional group
Gp-Usg: Real-time ultrasound-guided group
LOR: Loss of resistance
SD: Standard deviation
TEA: Thoracic epidural analgesia
TEP: Thoracic epidural placement
USG: Ultrasound-guided
